# The neural signature of high myopia: structural and functional brain alterations and their cognitive-emotional associations

**DOI:** 10.3389/fcell.2025.1634553

**Published:** 2025-08-05

**Authors:** Xuan Li, Kuan Zhao, Boling Deng, Zhengzheng Wu, Ling Wei

**Affiliations:** ^1^ Department of Ophthalmology, Sichuan Provincial People’s Hospital, University of Electronic Science and Technology of China, Chengdu, China; ^2^ Genetic Diseases Key Laboratory of Sichuan Province, Department of Medical Genetics, Department of Laboratory Medicine, Sichuan Academy of Medical Sciences & Sichuan Provincial People’s Hospital, School of Medicine, University of Electronic Science and Technology of China, Chengdu, China; ^3^ Department of Psychological Medicine, Fudan University Shanghai Cancer Center, Shanghai, China; ^4^ Department of Oncology, Shanghai Medical College, Fudan University, Shanghai, China; ^5^ Research Unit for Blindness Prevention of Chinese Academy of Medical Sciences (2019RU026), Sichuan Academy of Medical Sciences, Chengdu, China

**Keywords:** myopia, brain functions, cognition, emotion, cortical thinning

## Abstract

Beyond refractive error, myopia is increasingly recognized as a systemic condition with neurological implications, associated with visual dysfunction and structural retinal–choroidal alterations. This review synthesizes neuroimaging evidence demonstrating widespread neuroanatomical and functional brain changes in myopia, including cortical thinning, white matter disorganization, and disrupted functional connectivity, which may be associated with changes in cognitive-emotional systems rather than just the visual system. Mechanistically, these neural signatures reflect experience-dependent neural plasticity, dopaminergic dysregulation in the retinal ON pathway, and non-image-forming disruptions mediated by intrinsically photosensitive retinal ganglion cells, compounded by vascular dysfunction and impaired neurovascular coupling. Clinically, these findings highlight the importance of early neurocognitive risk assessment through multimodal imaging and psychological screening. By elucidating the retina–brain axis, this review bridges ophthalmological and neurological perspectives, guiding precision interventions for comprehensive, life-course myopia management.

## 1 Introduction

Myopia, the most common type of refractive error, is characterized by light being focused in front of the retina, resulting in blurred distance vision (Baird et al., 2020). Myopia has emerged as one of the leading causes of visual impairment worldwide, currently affecting nearly 2 billion people globally (Baird et al., 2020). It is estimated that nearly half of the world’s population will be myopic by 2050 (Liang et al., 2025), posing a growing public health concern due to the risk of sight-threatening complications.

Visual processing accounts for a substantial portion of brain activity, engaging diverse cortical and subcortical regions that decode, integrate, and respond to visual stimuli. The integrity of this system depends on both optical clarity and accurate neural transmission from the retina to higher visual centers. Myopia, especially high myopia, is frequently often accompanied by retinal ischemia and hypoxia, chorioretinal thinning and atrophy, and also pathological damages to retinal ganglion cells, which are the primary conduits of visual signals to the central nervous system (Wu Q. et al., 2020). On the other hand, myopic eyes, even with spectacle-corrected visual acuity, still exhibit impairments in higher-order visual functions that rely on complex brain processing, including reduced contrast sensitivity (Kerber et al., 2016; Wei et al., 2022), stereoacuity (Yang et al., 2013; Guo et al., 2016), visual attention (Turatto et al., 1999), and visual-motor integration (Findlay et al., 2020). In light of accumulating neuroimaging and clinical evidence, myopia-related structural and functional alterations might degrade both the quality and quantity of input to central visual circuits, potentially leading to downstream neural consequences across visual and associative regions. Therefore, anatomical and functional disruptions in myopia should be regarded not merely as ocular conditions but as indicators of broader neural vulnerability.

**FIGURE 1:**
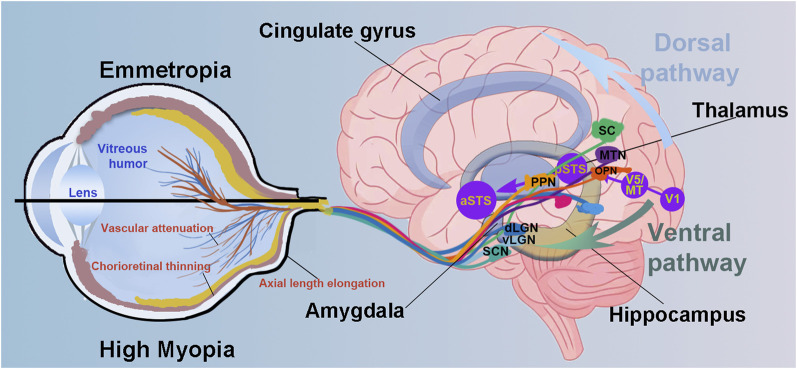
Schematic diagram illustrating eye–brain pathways and potential neural alterations associated with high myopia. Compared to emmetropic eyes, highly myopic eye presents the axial length elongation, chorioretinal thinning and atrophy and vascular attenuation. Visual information is transmitted via retinal ganglion cells to multiple subcortical and cortical regions through parallel pathways. The diagram emphasizes both the dorsal pathway (involved in spatial attention and visuomotor integration) and the ventral pathway (related to object recognition and emotional processing). Key visual and non-image-forming areas are also illustrated. Altered input from the retina in high myopia may disrupt downstream processing in these brain regions, associating with potential deficits in visual perception, cognition, and emotion. Suprachiasmatic nucleus, SCN; dorsal and ventral lateral geniculate nucleus, dLGN and vLGN; superior colliculus, SC; primary visual cortex, V1; pedunculopontine nucleus, PPN; olivary pretectal nucleus, OPN; medial terminal nucleus, MTN; middle temporal visual area, V5/MT; posterior superior temporal sulcus, pSTS and aSTS, posterior and anterior superior temporal sulcus.

Here, we review the current evidence on the neural signature of high myopia, focusing on alterations in brain structure and function and their cognitive-emotional impact (Figure 1). We advocate for a life-course management approach of myopia that goes beyond vision correction to include early screening for neural complications, monitoring of cognitive and emotional health, and the development of neuroprotective strategies aimed at preserving both visual and brain function, ultimately enhancing quality of life for individuals with myopia.

## 2 Neuroanatomical alterations in myopia

### 2.1 Whole-brain changes

Recent neuroimaging studies have shown that high myopia is linked to specific changes in global brain structures. While Li et al. found no significant differences in gray matter concentration, they reported increased white matter concentration in the calcarine cortex of high myopia patients (Li et al., 2012). Additionally, a large-scale UK Biobank analysis linked myopia (≤−0.75 D) to reduced total brain and white matter volumes (WMV), but not gray matter volume (GMV), although Mendelian randomization did not support a causal relationship (Wei Zhang et al., 2025). In contrast, other studies using voxel-based morphometry method have revealed broader structural changes with GMVs. Huang et al. observed reduced whole-brain GMV and total intracranial volume in highly myopic individuals (Huang et al., 2018). Similarly, form-deprivation myopic rats exhibited decreased GMV in several regions, including the primary and secondary visual cortices, subiculum, cornu ammonis, entorhinal cortex, and cerebellar molecular layer (Animal study: Liu et al., 2023). Collectively, these findings suggest that myopia, particularly high myopia, is associated with widespread changes in brain structures, notably in white matter concentration and volume, while gray matter changes appear more variable across imaging modalities and analytic methods.

### 2.2 Regional brain changes

Recent studies have shown that myopia is linked to distinct regional alterations in brain morphology, particularly in areas involved in visual and sensorimotor processing. Wu et al. reported reduced cortical thickness in the primary visual cortex (V1) and primary motor cortex (M1), along with increased thickness in the parietal operculum (OP4), a region associated with tactile processing, suggesting action-dependent neuroplasticity in response to altered visual experience (Wu Y. J. et al., 2020). Similarly, Huang et al. found significant GMV reductions in the right cuneus/lingual gyrus and right thalamus, regions crucial for visual information integration and processing (Bogousslavsky et al., 1987; Vanni et al., 2001; Mirzajani et al., 2011; Usrey and Alitto, 2015). Collectively, these findings indicate that the visual cortex and associated regions may undergo adaptive or maladaptive structural remodeling in response to the occasional blurred retinal input in high myopia.

### 2.3 White matter tract alterations

White matter tracts are essential for facilitating efficient neural communication across brain regions. In high myopia, growing evidence suggests that specific pathways are particularly susceptible to microstructural disruption. Significantly reduced fractional anisotropy (FA) were found in several major tracts among high myopia patients (Wang et al., 2021), including the bilateral corticospinal tract, right inferior longitudinal fasciculus, superior longitudinal fasciculus, inferior fronto-occipital fasciculus, and left thalamus. These tracts are known to support motor conduction and higher-order visual processing (Parks and Madden, 2013; Zhang et al., 2015). The observed FA reductions imply that axonal damage may contribute to the underlying microstructural alterations in individuals with myopia (Wu and Cheung, 2010; Wang et al., 2018). These findings underscore the potential role of widespread white matter abnormalities in the neuropathophysiological of myopia, extending its impact beyond the visual cortex to broader neural networks.

### 2.4 Retina–brain structural associations

Recent advances in multimodal imaging have enabled researchers to investigate the relationship between retinal microstructure, assessed by optical coherence tomography (OCT), and brain morphology measured through magnetic resonance imaging (MRI). High myopia is associated with reduced peripapillary retinal nerve fiber layer (pRNFL) thickness (Tai et al., 2018; Lee et al., 2019), which has been linked to structural atrophy in the cingulate cortex and to episodic memory decline in older adults (Shi et al., 2019). Furthermore, Huang et al. found that RNFL thinning correlated with reduced GMV in the bilateral parahippocampal gyrus and thalamus, areas involved in memory and visual relay (Huang et al., 2018), suggesting that retinal degeneration may mirror central brain changes in high myopia.

## 3 Brain functional alterations in myopia

### 3.1 Abnormalities in neuro-visual functions

Despite spectacle-corrected visual acuity, myopic patients still exhibit deficits in visual perception and visuomotor coordination compared to emmetropes.

Significant declines in contrast sensitivity at higher spatial frequencies have been reported in individuals with high and severe myopia, even wearing best-corrected spectacle lenses (Liou and Chiu, 2001), with the severity worsening as axial length increased (Wei et al., 2022). Additionally, myopes (defined by SE from −0.50 to −14.00D) show a more pronounced decrease in contrast sensitivity in the far periphery when their attention is focused on the central vision as compared to emmetropes (Kerber et al., 2016). Furthermore, it’s also reported that highly myopic patients experience deficits in motion perception and blue-yellow contrast perception (García-Domene et al., 2018; Kuo et al., 2018).

Stereopsis, the brain’s capacity to perceive depth from binocular disparity, is a vital aspect of visual function and is reported to be disrupted in individuals with myopia. Emerging evidence indicates that such deficits stem not only from optical blur but also from deeper impairments in binocular integration in brain. A cross-sectional study reported that individuals with myopia (SE from −0.75 to −8.00D) exhibited poorer stereopsis with flickering stimuli and greater binocular imbalance at high spatial and low temporal frequencies compared to emmetropes (Vera-Diaz et al., 2018). Similarly, Liu et al. also reported significantly poorer stereopsis in high myopia than in moderate-to-low myopia (Liu et al., 2021). Accurate stereopsis requires precise binocular alignment, which can be assessed using perceptual eye position (PEP). Studies report that PEP is often impaired in high myopia. In particular, vertical PEP at 1° eccentricity correlates with myopia severity and poor stereopsis, highlighting the role of subtle vertical misalignment in binocular dysfunction (Xiao et al., 2025).

Eye movements, including saccades, smooth pursuit, fixation, accommodation, and vestibulo-ocular reflexes, contribute to the clarity and stability of retinal images (Agarwal et al., 2016). Increased axial length has been linked to reduced fixation stability (Zhu et al., 2019) and slower visually evoked saccadic eye movements (Müller et al., 2003). The cerebellum, a key structure for oculomotor control and visual-motor precision, plays a central role in coordinating these functions. Using three-dimensional pseudocontinuous arterial spin labeling, Wang et al. found significantly increased cerebral blood flow in the bilateral cerebellum of individuals with high myopia (Wang et al., 2020), suggesting a compensatory response to visual-motor dysfunction (Nitschke et al., 2004; Agarwal et al., 2016).

Collectively, the compromised contrast sensitivity, stereopsis, and eye movement dysfunction observed in high myopia may not only be influenced by peripheral structural factors, such as reduced photoreceptor density or diminished stimulation of magnocellular pathways, but also reflect underlying changes in spatiotemporal processing within the visual system. These findings underscore the importance of considering dynamic visual pathways in both clinical assessment and research to fully understand the neuro-visual impact of myopia.

### 3.2 Brain network dysfunctions

Recent neuroimaging studies reveal widespread brain network abnormalities in individuals with myopia. Compared with controls, highly myopic patients show altered regional brain activity, with reduced amplitude of low-frequency fluctuation (ALFF) in areas such as the temporal, frontal, and parietal cortices, and increased ALFF in regions like the midcingulate cortex and precuneus, areas involved in attention and sensory processing (Huang et al., 2016; Zhang et al., 2023). These changes suggest disruptions in spontaneous neural activity that may underlie deficits in attention and visual-motor coordination.

Network-level metrics further highlight impaired connectivity in high myopia. Decreased degree centrality (DC) has been reported in the fusiform gyrus and cingulum, regions linked to face recognition, memory, and emotion, indicating reduced network integration in pathological myopia (Zuo et al., 2012). Decreased DC in the medial frontal gyrus and inferior parietal lobule in high myopia patients also points to potential impairments in reading, language, and cognitive functions (Cheng et al., 2020). Decreased short- and long-range functional connectivity density in default mode network (a brain network involved in self-referential and cognitive processing) and cognitive control regions such as the posterior cingulate cortex, rostrolateral prefrontal cortex, and inferior temporal gyrus, reinforcing the link between high myopia and attentional deficits (Zhai et al., 2016).

Advanced metrics like dynamic regional homogeneity (dReHo) and voxel-mirrored homotopic connectivity (VMHC) further support disrupted functional coordination in high myopia. Highly myopic patients show increased dReHo in various regions involved in language, cognition, and sensorimotor processing (Ji et al., 2022a), alongside reduced VMHC in the putamen and fusiform gyrus, indicating decreased interhemispheric synchrony in motor and language networks (Cheng et al., 2022). Moreover, weakened connectivity within basal ganglia circuits and altered interactions between the salience and sensorimotor networks highlight broader disruptions (Cheng et al., 2022). Dysfunction within the default mode network and cerebellar network (regulates motor coordination and cognitive integration) further implicates high myopia in impairments across visual, cognitive, and motor domains (Ji et al., 2022b; Wei et al., 2024).

### 3.3 Brains attentional alterations and their visual effects

High myopia may also influence higher-order neural processing, particularly in attentional allocation and visual perception. A pivotal study using acuity discrimination tasks across different eccentricities demonstrated that individuals with low-to-moderate myopia exhibit narrower attentional windows and reduced attentional efficiency, especially in the peripheral visual field, compared to emmetropes (De Lestrange-Anginieur et al., 2025). Consistently, Kerber et al. reported that myopic individuals (SE from −0.50 to −14.00 D) showed greater deficits in peripheral contrast sensitivity when central attentional load was increased (Kerber et al., 2016). These behavioral findings suggest that myopia may induce a compensatory reallocation of cognitive resources, likely due to chronic reliance on central fixation and degradation of peripheral input. Supporting this, neuroimaging studies have revealed abnormal variability in visual sensorimotor and attention-related brain regions in individuals with high myopia, implying possible reorganization within the attentional control network (Zhang et al., 2023).

### 3.4 Cognitive associations

Accumulating evidence suggests a potential association between myopia and cognitive dysfunction, particularly in older adults. A cross-sectional study of 1,032 Malay individuals aged 60 to 79 found that myopic participants (SE < −0.5D) were nearly twice as likely to exhibit cognitive dysfunction compared to emmetropic ones (Ong et al., 2013). Similarly, a large-scale study of 4,123 Chinese adults aged ≥60 reported a similar 2-fold increased risk of cognitive dysfunction among myopic individuals (SE < −0.5D) (Sun et al., 2016). In contrast, a prospective German study using the Tower of London task reported better cognitive performance in myopic (SE ≤ −0.5 D) participants, but this association disappeared after adjusting for confounders such as education level (Mirshahi et al., 2016).

Beyond older populations, cognitive deficits have also been observed in younger individuals with myopia. In a study of individuals aged 18–40, those with bilateral myopia (SE ≤ 0.5 D) showed poorer performance on tests of processing speed and episodic memory compared to matched controls (Hussein et al., 2023). Particularly, cognitive scores correlated with both refractive error severity and RNFL thinning, suggesting that structural retinal changes may be linked to cognitive decline, even in early adulthood (Hussein et al., 2023).

### 3.5 Emotional associations

Beyond visual impairment, structural and inflammatory changes linked to myopia may also contribute to emotional dysregulation, including depression and anxiety. A recent study reported elevated anxiety levels in individuals with high myopia (Zhu et al., 2023). Another study during the COVID-19 pandemic reported higher anxiety scores increase as myopic severity increased in Chinese university freshmen with myopia (Zhang et al., 2021). Similarly, individuals with high myopia, particularly young women ones (Osuagwu et al., 2023), were more likely to report poor physical health and clinically relevant depression (Guo et al., 2020).

In these mice models of myopia, increased circulating CC chemokine ligand 2 and monocytes infiltration, blood-brain barrier disruption, and alterations in dendritic spine density were observed within the basolateral amygdala and ventral hippocampus, regions closely associated with anxiety and depression (Animal study: Zhang et al., 2019; Wz et al., 2020; Zhu et al., 2023; Asim et al., 2024). These findings support a potential connection between inflammation, blood-brain barrier disruption, and anxiety in individuals with high myopia.

Neuroimaging studies have linked emotion-related brain regions to the emotional disturbances seen in high myopia. Cheng et al. found that individuals with high myopia had a significantly higher diffusion coefficient in the right parahippocampal gyrus compared to those with low myopia, suggesting microstructural changes (Cheng et al., 2020). Given this region’s role in emotion, memory, and visual processing, these findings suggest that high myopia may involve certain disruptions in brain circuits integrating visual and emotional functions.

Most current studies link high myopia to anxiety and depression, likely because these are the most commonly studied emotional disorders. However, since emotion-related brain regions like the amygdala and hippocampus are involved, other emotional changes—such as anhedonia, emotional reactivity, or social withdrawal—may also occur. Future research using broader emotional assessments could help reveal the full range of affective impacts related to myopia.

## 4 Potential mechanisms underlying neural changes in myopia

### 4.1 Visual deprivation and experience-dependent neural plasticity

Visual input is a fundamental driver of neural development and plasticity. In myopia, chronic degradation of visual signals due to retinal and axial elongation may relate to widespread structural and functional brain changes. These changes likely involve interrelated mechanisms such as cortical reorganization, neurotransmitter imbalance, and disrupted photic signaling.

#### 4.1.1 Cortical atrophy and reorganization

Irreversible fundus changes in high myopia, such as chorioretinal atrophy and retinal thinning, result in persistent visual input loss. This deprivation may contribute to cortical atrophy in visual-related areas and trigger compensatory reorganization in neural circuits. This impaired input can disrupt perceptual processing, and, in turn, impact higher-order cognitive functioning (Li et al., 2022).

#### 4.1.2 ON pathway and dopaminergic dysregulation

Reduced activation of the ON visual pathway, often due to prolonged near work in dim environments, contributes to myopia progression (Wilmet et al., 2024). This under-activation decreases dopamine release from amacrine cells, facilitating axial elongation (Animal studies: Iuvone et al., 1991; Chakraborty et al., 2015). Neuroimaging evidence further shows that GMV alterations in high myopia correlate with receptor densities for dopamine, serotonin, and GABA, suggesting broader neurochemical remodeling (Zhang et al., 2025).

#### 4.1.3 ipRGC pathways and cognitive-emotional links

Animal studies have shown that photosensitive retinal ganglion cells (ipRGCs) play a causal role in myopia development. Liu et al. reported that form-deprivation myopia is attenuated in ipRGC-ablated and melanopsin-deficient animals, while form-deprived eyes exhibit enhanced melanopsin expression and photoresponses (Liu et al., 2022). Similarly, melanopsin-expressing RGCs were proved to modulate myopia progression via dopaminergic mechanisms (Animal study: Chakraborty et al., 2022). Intrinsically ipRGCs project to the suprachiasmatic nucleus in the hypothalamus, which regulates circadian rhythms and influences cognitive function, as well as to the perihabenular nucleus in the dorsal thalamus, a region involved in mood regulation (Fernandez et al., 2018). Another recent study also demonstrated that ipRGCs project to limbic brain regions and contribute to cognitive-emotional alternations (Bedrosian et al., 2013).

### 4.2 Vascular contributions to neural dysfunction

Retinal microvascular compromise is a hallmark of high myopia. Wu et al. observed significant thinning of the retinal and choroidal layers, along with reduced retinal vessel density, in highly myopic eyes compared to the emmetropic or low-myopic controls (Wu Q. et al., 2020). These vascular changes are paralleled by thinning of the RNFL, which has been linked to reduced GMV This finding is relevant in the context of previous research, which has been linked to reduced GMV changes in visual processing areas of the brain (Huang et al., 2018).

Extending this brain-eye connection, Zhang et al. identified abnormal neurovascular coupling in individuals with high myopia, particularly in primary and higher-order visual cortices and regions involved in object and category recognition (Zhang et al., 2024). Neurovascular coupling, the mechanism that aligns cerebral blood flow with neural activity, was found to correlate with both refractive error and best-corrected visual acuity, suggesting that impaired vascular responsiveness may contribute to central functional deficits (Liang et al., 2013).

Together, these findings indicate that disrupted neurovascular balance is a key mechanism linking myopia to structural and functional brain alterations.

## 5 Discussion

Myopia, particularly high myopia, is increasingly recognized as a systemic condition with neurological implications beyond visual impairment. Recent studies associate myopia with structural and functional brain alterations, cognitive deficits, and emotional disturbances. In China, alarming data reveal that over 70% of myopic primary school students lack proper refractive correction (Wang et al., 2023), exacerbating axial elongation and compromising visual development. While uncorrected myopia poses a critical public health challenge in children, even corrected myopia carries risks in older adults due to systemic complications. These findings underscore the urgent need for myopia control strategies that address both refractive correction and neurocognitive protection to mitigate the condition’s broader cognitive-emotional burden.

Emerging evidence demonstrates that myopia-related neurological alterations manifest through RNFL thinning, impaired neurovascular coupling, and cortical atrophy, all established biomarkers of cognitive and emotional dysregulation (Li et al., 2022; Osuagwu et al., 2023). These findings have important clinical implications. First, the incorporation of OCT and brain MRI into standard clinical evaluations could enable earlier detection of neurodegenerative changes in myopic patients. Second, routine screening for cognitive and emotional symptoms should be considered in high-risk elder myopic populations. Third, long-term management strategies should incorporate both lifestyle modifications (e.g., increased outdoor activity, visual and cognitive training) and targeted neuroprotective approaches to mitigate potential central nervous system complications.

While we focused primarily on high myopia or pathological myopia, questions remain regarding the extent to which these neural alterations are present in low-to-moderate myopia. Some studies have reported that the severity of myopia (refractive error or axial length) correlates with the magnitude of deficits in visual function, attentional processing, or brain morphology (Zhang et al., 2021; Osuagwu et al., 2023), suggesting a possible continuum of neural adaptation or vulnerability across the myopia spectrum. Additionally, retinal imaging biomarkers such as RNFL thickness and macular parameters, which may reflect early stages of myopic progression, have been associated with structural and functional traits in the brain, particularly within the primary visual cortex and visual pathways (Zhao et al., 2024).

In conclusion, elucidating the neurostructural and functional alterations associated with myopia may reveal novel neuromodulatory targets for early intervention in myopia-related cognitive and emotional comorbidities. The accumulating evidence underscores the potential for developing precision therapeutic approaches that integrate ocular and neural protection strategies. These findings advocate for a paradigm shift toward comprehensive, life-course management of myopia that addresses both its ocular and central nervous system manifestations.
